# Failure to maintain full-term pregnancies in pig carrying *klotho* monoallelic knockout fetuses

**DOI:** 10.1186/s12896-020-00660-9

**Published:** 2021-01-07

**Authors:** Sanghoon Lee, Min Hee Jung, Kilyoung Song, Jun-Xue Jin, Anukul Taweechaipaisankul, Geon A. Kim, Hyun Ju Oh, Ok Jae Koo, Se Chang Park, Byeong Chun Lee

**Affiliations:** 1 Department of Theriogenology and Biotechnology, College of Veterinary Medicine, Seoul National University, Seoul, Republic of Korea; 2 Futuristic Animal Resource & Research Center, Korea Research Institute of Bioscience and Biotechnology, Cheongju-si, Chungcheongbuk-do Republic of Korea; 3 Toolgen, Inc., Seoul, Republic of Korea; 4 Key Laboratory of Animal Cellular and Genetic Engineering of Heilongjiang Province, College of Life Science, Northeast Agricultural University, Harbin, Heilongjiang China; 5 Department of Biomedical Laboratory Science, School of Medicine, Eulji University, Daejeon, Republic of Korea; 6 Laboratory of Aquatic Biomedicine, College of Veterinary Medicine and Research Institute for Veterinary Science, Seoul National University, Seoul, Republic of Korea

**Keywords:** CRISPR/Cas9, Somatic cell nuclear transfer, *Klotho*, Aging, Pregnancy loss

## Abstract

**Background:**

Small animals that show a deficiency in klotho exhibit extremely shortened life span with multiple aging-like phenotypes. However, limited information is available on the function of klotho in large animals such as pigs.

**Results:**

In an attempt to produce *klotho* knockout pigs, an sgRNA specific for *klotho* (targeting exon 3) was designed and Cas9-sgRNA ribonucleoproteins were transfected into porcine fibroblasts. Transfected fibroblasts were cultured for one to 2 days and then directly used for nuclear transfer without selection. The cloned embryos were cultured in vitro for 7 days and analyzed to detect modifications of the *klotho* gene by both T7E1 and deep sequencing analysis. Modification succeeded in 13 of 20 blastocysts (65%), 8 of which (40.0%) were monoallelic modifications and 5 (25.0%) were biallelic modifications. Based on high mutation rates in blastocysts, we transferred the cloned embryos to 5 recipient pigs; 1 recipient was pregnant and 16 fetuses were recovered at Day 28 post transfer. Of the 16 fetuses, 9 were resorbing and 7 were viable. Four of 9 (44.4%) resorbing fetuses and 3 of the 7 (42.9%) viable fetuses had monoallelic modifications. Thus, 3 *klotho* monoallelic knockout cell lines were established by primary culture. A total of 2088 cloned embryos reconstructed with 2 frame-shifted cell lines were transferred to 11 synchronized recipients. Of the recipients, 7 of 11 eleven (63.6%) became pregnant. However, none of the pregnancies was maintained to term. To discover why *klotho* monoallelic knockout fetuses were aborted, expression of aging- and apoptosis-related genes and klotho protein in placentas from *klotho* monoallelic knockout and wild-type fetuses was investigated. Placentas from *klotho* monoallelic knockout fetuses showed negatively changed expression of aging- and apoptosis-related genes with lower relative expression of klotho protein. These results indicated that the reason why *klotho* monoallelic knockout fetuses were not maintained to term was possibly due to decreased klotho expression in placentas, negatively affecting aging- and apoptosis-related genes.

**Conclusions:**

*Klotho* monoallelic knockout porcine fetal fibroblasts were successfully established. However, pigs carrying *klotho* monoallelic knockout fetuses failed to maintain full-term pregnancy and a decrease in klotho expression in placenta likely leads to pregnancy loss.

**Supplementary Information:**

The online version contains supplementary material available at 10.1186/s12896-020-00660-9.

## Background

Genetically engineered pigs are useful models for studying human diseases due to the similarity of their anatomy and physiology to those of humans [1]. Recent advances in genome editing techniques such as Zinc-Finger nucleases (ZFNs), Transcription activator-like effector nucleases (TALENs) and the clustered regularly interspaced short palindromic repeat (CRISPR)/CRISPR-associated (Cas9) system have enabled the production of animal models for specific purposes [2]. In particular, recent application of the CRISPR/Cas9 system has improved genome editing mutation efficiency compared to previous meganucleases (ZFNs and TALENs) [3].

CRISPR-mediated genome editing in pigs is generally accomplished by somatic cell nuclear transfer (SCNT) with donor cells transfected with single-guide RNA (sgRNA) and Cas9 DNA [4]. However, transfection with plasmid DNA encoding sgRNA and Cas9 is limited by off-target effects and unwanted integration of DNA segments at both on-target and off-target sites in the genome [5]. To overcome this issue, delivery of Cas9-sgRNA ribonuleoproteins (RNPs) into cells or embryos has been used. Furthermore, delivery of pre-assembled Cas9-sgRNA RNPs facilitates highly efficient genome editing in cells, embryos, and organisms [6–8].

The klotho deficient mice due to a defect in *klotho* gene expression display multiple aging-like phenotypes similar to human premature-aging syndromes [9]. They develop normally until 3 weeks of age, but soon after begin to show multiple aging-like phenotypes such as growth retardation, infertility, arteriosclerosis, osteoporosis, and finally premature death at 2 months of age.

Although *klotho* knockout mice exhibit multiple aging-like phenotypes, pigs may be a more suitable animal model for human aging [10]. However, studies using large animal models such as pigs have been limited due to the absence of cell lines or animal models. Therefore, the objective of this study was to establish *klotho* knockout cell lines and to produce cloned pigs by nuclear transfer of *klotho* knockout fetal fibroblasts.

## Results

### Evaluation of preimplantation embryo development and genome editing efficiency after SCNT using non-selected donor cells transfected with *klotho*-targeting Cas9-sgRNA RNPs

**Fig. 1:**
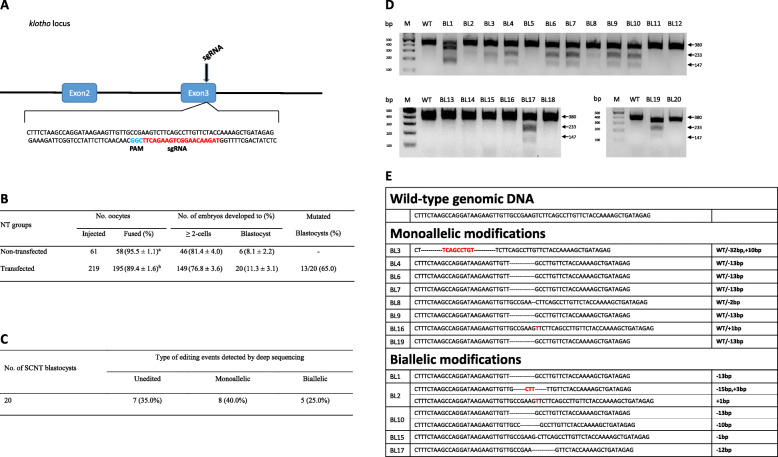
Generation of *klotho* gene knockout blastocysts by SCNT using non-selected porcine fibroblasts transfected with Cas9-sgRNA RNPs. **a.** Schematic representation of sgRNA specific to exon 3 of the porcine *klotho* locus. The sgRNA targeting sequence is highlighted in red and protospacer adjacent motif (PAM) is highlighted in blue. **b.** Comparison of preimplantation development of cloned embryos after SCNT with Cas9-sgRNA RNPs (targeting the *klotho* gene) transfected or non-transfected fibroblasts. Within each category, groups marked with different letters are significantly different (*P* < 0.05). **c.** Rate of DNA editing on the *klotho* gene of blastocysts generated by SCNT using non-selected porcine fibroblasts transfected with Cas9-sgRNA RNPs. **d.** T7 endonuclease I (T7E1) assay: the T7E1 assay was conducted using genomic DNA from 20 cloned blastocysts. (M, Marker; WT, wild-type; BL, blastocyst). For improved clarity and conciseness, cropped areas of the gel are shown. All cropped regions originate from the same gel (Additional file 2: Fig. S1). **e.** A diagram illustrating the editing scheme on exon 3 of the *klotho* gene of blastocysts

In order to investigate the effect of direct use of Cas9-sgRNA RNPs-transfected donor cells for SCNT on embryo development, we compared the preimplantation development of embryos after SCNT with Cas9-sgRNA RNPs (targeting the *klotho* gene) transfected or non-transfected fibroblasts (Fig. 1b). We made a total of 253 SCNT embryos using transfected (*n* = 195) and non-transfected donor cells (*n* = 58), which developed into 20 and 6 blastocysts, respectively. There was no significant difference in cleavage and blastocyst formation rates; however, there was a significant difference in fusion rates between the transfected and non-transfected groups (89.4 ± 1.6 vs. 95.5 ± 1.1). In the T7E1 assay, 13 of 20 (65%) blastocysts that were derived from Cas9-sgRNA RNPs transfected cells showed modifications in the *klotho* gene (Fig. 1d). To confirm the modifications, we performed deep sequencing analysis and the result showed that 8 blastocysts contained monoallelic modifications (40.0%) and 5 blastocysts contained biallelic modifications (25.0%) (Fig. 1c and e).

### Evaluation of genome editing efficiency after embryo transfer and establishment of *klotho*-knockout fetal cell lines

**Fig. 2:**
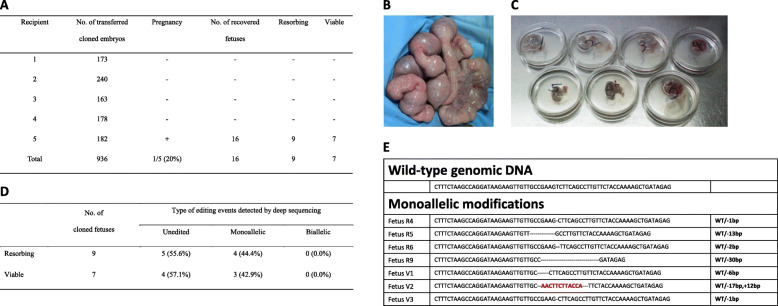
Generation of *klotho* gene knockout fetuses by transfer of embryos cloned from non-selected porcine fibroblasts transfected with Cas9-sgRNA RNPs. **a.** Results of transfer of cloned embryos reconstructed with non-selected porcine fibroblasts transfected with Cas9-sgRNA RNPs (targeting the *klotho* gene) to fertile recipients. **b.** The uterus of the pregnant recipient and **c.** the 7 viable fetuses at day 28 of gestation. **d.** Rate of DNA editing on the *klotho* gene of fetuses recovered after 28 days post embryo transfer. **e.** A diagram illustrating the editing scheme for exon 3 of the *klotho* gene of fetuses generated by SCNT using non-selected porcine fibroblasts transfected with Cas9-sgRNA RNPs. Fetus R, resorbing fetus; Fetus V, viable fetus

Based on high modification rates in preimplantation embryos, we performed embryo transfer after SCNT with non-selected donor cells transfected with *klotho*-targeting Cas9-sgRNA RNPs. We transferred a total of 936 SCNT embryos to 5 recipients, 1 of which became pregnant (Fig. 2a). After 28 days, 16 cloned fetuses were recovered, 9 of which were resorbing and 7 were viable (Fig. 2b and c). We performed primary culture using body parts of viable fetuses to establish *klotho*-knockout fetal cell lines (Fetus V1–3) and carried out deep sequencing using the remaining tissue of the viable and resorbing fetuses. In deep sequencing analysis, 4 (44.4%) of 9 resorbing fetuses and 3 (42.9%) of 7 viable fetuses showed monoallelic modifications (Fig. 2d and e).

### Effects of *klotho* monoallelic knockout on gene expression in fetal fibroblasts and on preimplantation development of embryos cloned from this cell line

**Fig. 3:**
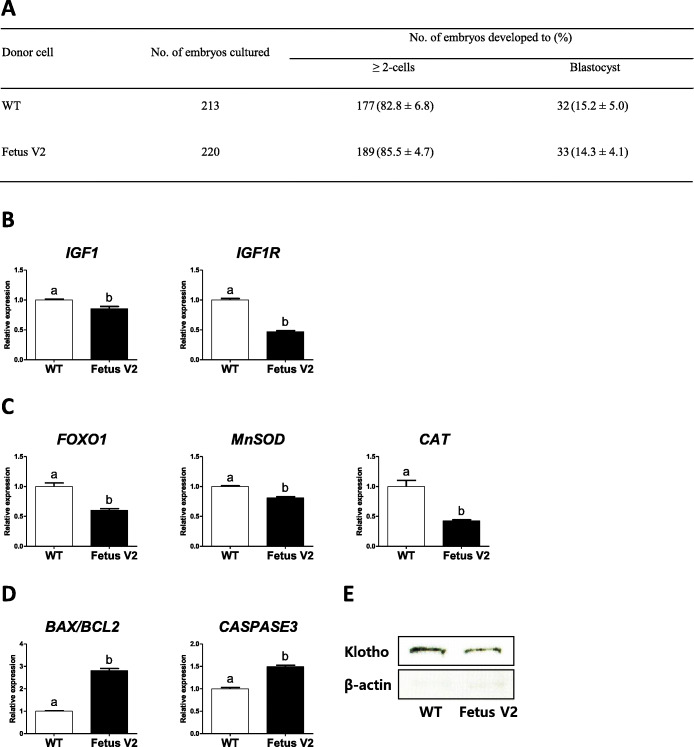
Effects of *klotho* monoallelic knockout on gene expression in fetal fibroblasts and on preimplantation development of embryos cloned from this cell line. **a.** Comparison of preimplantation development of the cloned embryos reconstructed with wild-type or *klotho* monoallelic knockout fetal fibroblasts. The experiment was replicated seven times. **b-e.** Comparison of expression of aging- and apoptosis-related genes and klotho protein between *klotho* monoallelic knockout and wild-type fetal fibroblasts. Wild-type fetal fibroblasts originated from the syngeneic cells that were used to add the *klotho* gene modifications. Wild-type and *klotho* monoallelic knockout fibroblasts were used for gene expression analysis (wild-type, *n* = 3; Fetus V2, *n* = 3). **b.** IGF1 signaling genes (*IGF1* and *IGF1R*). **c.** *FOXO1* and antioxidant genes (*MnSOD* and *CAT*). **d.** Apoptosis-related genes (*BAX*/*BCL2* ratio and *CASPASE3*). Within each category, groups marked with different letters are significantly different (*P* < 0.05). The experiment was replicated three times. **e.** Expression of klotho protein as detected by Western blot analysis. For improved clarity and conciseness, cropped areas of the blot are shown. All cropped regions originate from the same blot (Additional file 2: Fig. S2). WT, wild-type; Fetus V2, viable fetus 2 (WT/− 17 bp,+ 12 bp)

Gene expression in *klotho* monoallelic knockout fetal fibroblasts (Fetus V2) and wild-type fetal fibroblasts was compared. Wild-type fetal fibroblasts originated from the syngeneic cells that were used to add the *klotho* gene modifications. In this experiment, expression of genes related to aging (IGF1 signaling genes, *FOXO1,* and antioxidant genes) and apoptosis was evaluated. As shown in Fig. 3b, expression of *IGF1* and *IGF1R* was significantly decreased in the Fetus V2 fibroblasts. Additionally, Fetus V2 fibroblasts showed a significant decrease in expression of *FOXO1* and its downstream target genes with antioxidant function (*MnSOD* and *CAT*) (Fig. 3c). Regarding apoptosis-related genes, the *BAX*/*BCL2* ratio and expression of *CASPASE3* increased significantly in Fetus V2 fibroblasts (Fig. 3d). Moreover, we found a lower expression of klotho protein in the Fetus V2 fibroblasts (Fig. 3e). In order to investigate the effect of *klotho* monoallelic knockout on preimplantation embryo development, the developmental competence of cloned embryos reconstructed with wild-type or *klotho* monoallelic knockout fetal fibroblasts (Fetus V2) was evaluated. No significant difference was observed in cleavage and blastocysts formation rates between cloned embryos reconstructed with wild-type and Fetus V2 fibroblasts (82.8 and 15.2% vs. 85.5 and 14.3%, respectively) (Fig. 3a).

### Transfer of *klotho* monoallelic knockout cloned embryos to fertile recipients and comparison of expression of aging- and apoptosis-related genes and klotho protein in placentas

**Fig. 4:**
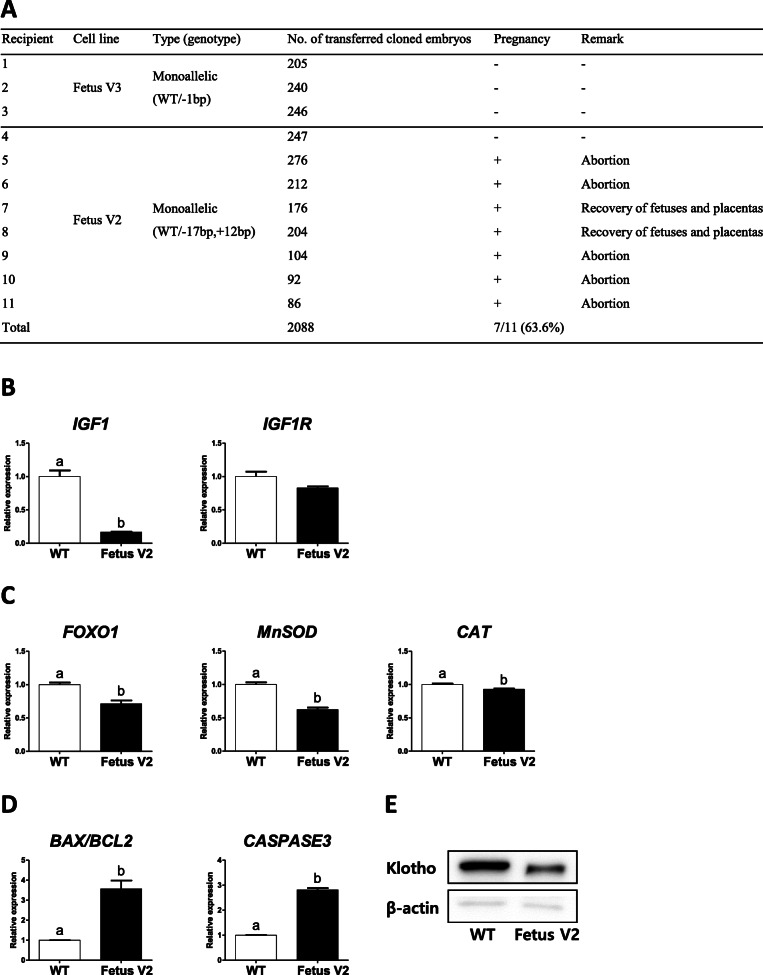
Failure to maintain pregnancy in recipients carrying *klotho* monoallelic knockout fetuses. **a.** Transfer of cloned embryos reconstructed with *klotho* monoallelic knockout fetal fibroblasts. **b-e.** Comparison of expression of aging- and apoptosis-related genes and klotho protein between *klotho* monoallelic knockout and wild-type placentas. Fetuses along with their placentas were recovered (wild-type, *n* = 12; Fetus V2, *n* = 3) between the fourth to fifth week after embryo transfer. Placentas were used for gene expression analysis (wild-type, *n* = 3; Fetus V2, *n* = 3). **b.** IGF1 signaling genes (*IGF1* and *IGF1R*). **c.** *FOXO1* and antioxidant genes (*MnSOD* and *CAT*). **d.** Apoptosis-related genes (*BAX*/*BCL2* ratio and *CASPASE3*). Within each category, groups marked with different letters are significantly different (*P* < 0.05). The experiment was replicated three times. **e.** Expression of klotho protein as detected by Western blot analysis. For improved clarity and conciseness, cropped areas of the blot are shown. All cropped regions originate from the same blot (Additional file 2: Fig. S3). WT, wild-type; Fetus V2, viable fetus 2 (WT/− 17 bp,+ 12 bp); Fetus V3, viable fetus 3 (WT/− 1 bp)

Based on the result showing normal preimplantation development of cloned embryos reconstructed with *klotho* monoallelic knockout fibroblasts, a total of 2088 cloned embryos reconstructed with Fetus V2 and V3 fibroblasts were transferred to 11 synchronized recipients (Fig. 4a). At day 25 after embryo transfer, 7 of 11 recipients (63.6%) were confirmed pregnant by ultrasonography. However, none of the pregnancies maintained to term. To discover why *klotho* monoallelic knockout fetuses were aborted, we recovered placentas from *klotho* monoallelic knockout fetuses and wild-type fetuses between the fourth to fifth week after embryo transfer. Fetuses along with their placentas were recovered (wild-type, *n* = 12; Fetus V2, *n* = 3). Placentas were used for gene expression analysis (wild-type, *n* = 3; Fetus V2, *n* = 3). Expression of aging- and apoptosis-related genes and klotho protein was investigated. Fetus V2 placentas showed significantly decreased expression of *IGF1*, *FOXO1* and downstream antioxidant genes (*MnSOD* and *CAT*), compared to wild-type placentas (Fig. 4b and c). In terms of apoptosis-related genes (Fig. 4d), the *BAX*/*BCL2* ratio and expression of *CASPASE3* increased significantly in Fetus V2 placentas. Furthermore, lower relative expression of klotho protein in the Fetus V2 placentas was confirmed (Fig. 4e).

### Off-target analysis

**Fig. 5:**
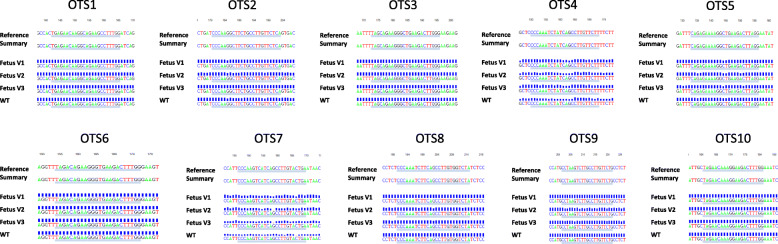
Sequencing results of 10 potential off-target sites (OTSs) for the sgRNA targeting porcine *klotho* gene. Direct sequencing of PCR products from wild-type and *klotho* monoallelic knockout fetal fibroblasts (Fetus V1, V2, and V3) using primers specific to 10 potential OTSs. WT, wild-type; Fetus V1, viable fetus 1 (WT/− 6 bp); Fetus V2, viable fetus 2 (WT/− 17 bp,+ 12 bp); Fetus V3, viable fetus 3 (WT/− 1 bp)

To test whether off-target events occurred in *klotho* monoallelic knockout fetal fibroblasts (Fetus V1, V2, and V3), 10 potential off-target sites (OTSs) for the sgRNA targeting porcine *klotho* gene were screened (Additional file 1: Table S1). The fragments around the potential OTSs were amplified by PCR and then sequenced. As shown in Fig. 5, none of the sequencing reads showed mutations, suggesting that no off-target events occurred at 10 potential OTSs in *klotho* monoallelic knockout fetal cell lines.

## Discussion

This study demonstrated *klotho* gene modifications in cloned fetuses generated by transfer of embryos reconstructed with non-selected Cas9-sgRNA transfected fetal fibroblasts. Additionally, *klotho* monoallelic knockout cell lines were successfully established from cloned fetuses and pregnancies of SCNT embryos reconstructed with these cell lines were confirmed. However, none of the pregnancies was maintained to term and we found that a decrease in klotho expression could lead to loss of pregnancy, possibly due to changes in expression of aging- and apoptosis-related genes in placentas.

For the successful production of genetically modified pigs, the establishment of early passage knockout cell lines are needed to overcome reduced preimplantation development of the cloned embryos that were reconstructed with high passage of knockout cell lines derived from a single colony. High passage number and longer period of in vitro culture are not suitable donor cell conditions for successful embryo development following SCNT [11]. To solve this problem, we investigated whether the use of non-selected donor cells transfected with Cas9-sgRNA RNPs for nuclear transfer results in high mutation rates in embryos and fetuses for establishment of *klotho* knockout fetal cell lines. Although there was a significant decrease in fusion rate of Cas9-sgRNA transfected group, it was a small decrease (89.4% vs. 95.5%) compared to the control group. The reason for this decrease might be due to the effect of transfection. Transfection of cells could alter cellular morphology, resulting in cells with a rough surface. Previously, it was reported that donor cells with a rough shape would influence the fusion rate of SCNT couplets [12]. Moreover, the fusion rate of SCNT embryos cloned from transfected cells with a rough surface showed lower fusion rate [13]. Nevertheless, SCNT using non-selected donor cells transfected with Cas9-sgRNA RNPs generated high mutation rates (65.0%) in blastocysts, without creating differences in cleavage and blastocyst formation rates compared to SCNT using wild-type fibroblasts. Based on high mutation rates and normal development in preimplantation embryos, we performed embryo transfer after SCNT with non-selected donor cells transfected with *klotho* targeting Cas9-sgRNA RNPs. After 28 days, 7 viable cloned fetuses were recovered from one pregnant recipient. Of the 7 viable fetuses, 3 (42.9%) showed monoallelic modifications and we used them to establish primary cell lines. SCNT with non-selected donor cells transfected with Cas9-sgRNA RNPs made it possible to establish early passage of *klotho* knockout cell lines derived from cloned fetuses. Then, we used 2 frame-shifted *klotho* monoallelic knockout cell lines as donor cells for production of cloned *klotho* knockout pigs.

After confirmation of normal preimplantation development of *klotho* monoallelic knockout embryos, cloned embryos reconstructed with 2 frame-shifted cell lines (Fetus V2 and V3) were transferred to 11 synchronized recipients. Pregnancies took place in 7 of 11 recipients (63.6%). Unfortunately, all of the 7 pregnant recipients carrying *klotho* monoallelic knockout fetuses were aborted at approximately the 5th week post transfer. Serial cloning might be a reason for their failure to maintain full-term pregnancies during the recloning experiments. Previously, Liu et al. suggested that serial cloning in pigs could compromise production efficiency due to the accumulation of developmental abnormalities [14]. However, we have successfully produced two kinds of genetically modified pigs using serial cloning method [15, 16]. Moreover, it was confirmed that failure to maintain full-term pregnancies was not off-target effect because there was no off-target alternation at potential OTSs in *klotho* monoallelic knockout fetal fibroblasts. Therefore, it is reasonable to assume that *klotho* monoallelic knockout might be the most likely reason for their failure to maintain full-term pregnancies.

To investigate the reason for these pregnancy losses, the relationship between klotho and pregnancy loss was studied and we speculated that these losses could be associated with reduced expression of klotho expression and oxidative stress. Recently, it was reported that the klotho expression levels in placenta is associated with preeclampsia [17], a disorder of pregnancy characterized by the onset of high blood pressure [18] and early pregnancy loss in severe cases [19]. Klotho mRNA and protein levels were reduced in preeclamptic placentas compared to controls [17]. Furthermore, as the anti-aging action of klotho contributed to regulation of oxidative stress by suppressing the insulin growth factor 1 (IGF1) signaling pathway [20, 21], reduced klotho expression may lead to oxidative stress in placenta. Klotho inhibits IGF1-induced autophosphorylation of the IGF1 receptor and subsequently it relieves the inhibitory effect of IGF1 signaling on Forkhead box O 1 (FOXO1), thereby inducing expression of downstream genes with antioxidant function (*MnSOD* and *CAT*) [22]. In this study, *klotho* monoallelic knockout fibroblasts and placentas showed decreased expression of genes related to IGF1 signaling, *FOXO1* and antioxidant function and increased expression of apoptotic genes. Therefore, the reason why pregnancies of recipients carrying *klotho* monoallelic knockout fetuses were not maintained to term may be due to reduced klotho expression in pregnant placenta and thereby negatively changing aging- and apoptosis-related genes in placentas.

Although *klotho* knockout mice were born [9], the present study indicated that recipient pigs carrying *klotho* monoallelic knockout fetuses were failed to maintain full-term pregnancies. It might be due to a difference in types of placenta between mice (hemochorial placenta) and pigs (epitheliochorial placenta). The placenta is a materno-fetal organ consisting of two components: the maternal placenta, which develops from the maternal uterine tissue, and the fetal placenta, which develops from the blastocyst that forms the fetus [23]. In hemochorial placenta of mice, the fetal chorion is in direct contact with the maternal blood vessel. However, in epitheliochorial placenta of pigs, the fetal chorion is in direct contact with the epithelium of the uterus, remaining separated from maternal blood vessel throughout gestation [24]. Therefore, failure to maintain full-term pregnancies in pig carrying *klotho* knockout fetuses might be due to difficulty in maternal compensation for the reduction of klotho placental expression.

## Conclusion

Early passage of *klotho* monoallelic knockout fetal fibroblasts were successfully established. In addition, cloned embryos reconstructed with these cell lines were produced and they showed normal preimplantation embryo development and implantation. However, recipients carrying *klotho* monoallelic knockout fetuses failed to maintain full-term pregnancy. A decrease in klotho expression in placentas negatively changed expression of aging- and apoptosis-related genes and it may be the cause of the observed loss of pregnancy.

## Methods

### Animals

The animals used in this study were maintained by the R&F farm (Boryeong, Korea) and the research farm of Gyeonggi Livestock and Veterinary Service (Osan, Korea). All animal experiments were reviewed and approved by the Institutional Animal Care and Use Committee of Seoul National University (IACUC approval number; SNU-160613-16) and performed in accordance with the Guide for the Care and Use of Laboratory Animals of Seoul National University.

### Design and construction of *klotho* targeting CRISPR/Cas9 system

The sgRNA that could recognize porcine *klotho* gene were designed using an online CRISPR design tool (http://zifit.partners.org/ZiFiT/Disclaimer.aspx). Sequence information of the designed sgRNA is 5′-TAGAACAAGGCTGAAGACTTCGG-3′. The protospacer adjacent motif (PAM) and sgRNA targeting sequence can be identified by the blue and red font, respectively (Fig. 1a). Specificity of the designed sgRNA was confirmed by searching for similar porcine sequences in GenBank. The sgRNA was designed to create double-strand breaks (DSBs) in exon 3 of *klotho*.

### Delivery of Cas9-sgRNA ribonucleoproteins

Cas9 protein in storage buffer (20 mM HEPES pH 7.5, 150 mM KCl, 1 mM DTT, and 10% glycerol) was mixed with sgRNA dissolved in nuclease-free water and incubated for 10 min at room temperature before use. To introduce DSBs in wild-type porcine fetal fibroblasts using ribonucleoproteins (RNPs), 9 × 10^5^ cells were transfected with Cas9 protein (28.8 μg) premixed with in vitro transcribed sgRNA (7.2 μg) through nucleofection (Neon; Invitrogen) with a 1400 V, 30 ms pulse width, and pulse number 1 setting. Transfected cells were subjected to a 4-well cell culture dish. After 1–2 days of transfection, non-selected cell population was directly used for somatic cell nuclear transfer.

### Somatic cell nuclear transfer (SCNT)

SCNT was performed as described in a previous study [25]. Briefly, oocyte manipulations were initiated at 40 h after in vitro maturation of oocytes. Enucleation was performed by aspirating the first polar body and adjacent cytoplasm containing the metaphase II chromosomes with an aspiration pipette. Then, a trypsinized fetal fibroblast with a smooth cell surface was transferred into the perivitelline space of an enucleated oocyte. These couplets were fused in a 20 μL droplet of fusion solution with a single DC pulse of 1.2 kV/cm for 30 μs using an electrical pulsing machine (LF101; NepaGene, Chiba, Japan). After 30 min, they were activated in activation solution with a single DC pulse of 1.5 kV/cm for 30 μs using a BTX Electro-Cell Manipulator 2001 (BTX, Inc., CA, USA). Electrically-activated embryos were cultured in Porcine Zygote Medium-5 (PZM-5; Funakoshi Corporation, Tokyo, Japan) at 39 °C in a humidified atmosphere of 5% O_2_, 5% CO_2_, and 90% N_2_ for 7 days.

### Embryo transfer and fetus recovery

At 1–2 days post-activation, 1- to 4-cell stage SCNT embryos were transferred into a naturally cycling recipient pig on day two after standing estrus was observed. A midventral laparotomy was performed under general anesthesia using isoflurane. The reproductive tract was exposed and the SCNT embryos (86–276 embryos) were transferred into both oviducts of a recipient pig. Pregnancy was diagnosed by ultrasonography on Day 25 (The day of embryo transfer was considered Day 0) and fetuses were recovered on Day 28 post transfer.

### Primary culture of porcine fetal fibroblasts

Under anesthesia through the placental passage of isoflurane, euthanasia of individual fetuses was induced by decapitation with surgical scissors. Euthanized fetus was dissected into three parts: head, body, and tail. Just the body parts of fetuses were washed three times in phosphate-buffered saline (PBS; Gibco, CA, USA) containing 1% Penicillin/Streptomycin (P/S; Gibco) and then chopped into small pieces in a 60 mm dish with Dulbecco’s Modified Eagle’s Medium (DMEM; Gibco). Well-dissociated tissues were centrifuged at 1500 rpm for 2 min. The supernatant was discarded, and the pellet was resuspended with DMEM and then centrifuged at 1500 rpm for 2 min. These procedures were repeated two times. Finally, the supernatant was discarded, and the pellet was resuspended in DMEM supplemented with 20% fetal bovine serum (FBS; Gibco), 1% P/S, 1% nonessential amino acid (NEAA; Gibco), and 100 mM β-mercaptoethanol (β-ME) by inverting the tube several times. The suspension was transferred into a cell culture dish for ~ 10 days with culture medium changed every 2–3 days. These primary cells were cultured, expanded, and frozen at − 196 °C for further use.

### T7E1 assay

Genomic DNA was extracted using Exgene TM cell SV (GeneAll Biotech., Seoul, Korea) according to the manufacturer’s instructions. PCR amplicons including CRISPR/Cas9 target sites were generated using the primers listed in Table 1. The T7E1 analysis was done as described previously (Kim et al., 2009). In brief, the PCR amplicons were denatured at 95 °C and annealed to form heteroduplex DNA, which was treated with 5 units of T7 endonuclease 1 (ToolGen Inc., Seoul, Korea) for 20 min at 37 °C and then analyzed by 2% agarose gel electrophoresis.

**Table 1:** Details of primers used for T7E1 assay and deep sequencing

Target	Use	Primer sequences (5′-3′)	Product size (bp)
*Klotho*	T7E1	F: CCTCAAGTAGTAAAACCCTC	379
		R: GGTTTTGTCAGCTGACTTAC	
	Deep sequencing	F: CTTGCTCTTGTCCTCTTTCC	282
		R: CAACAATTCCCCAAGCAAAG	

### Deep sequencing

The on-target regions were amplified from genomic DNA by the primers listed in Table 1 and used for library construction. Equal amounts of the PCR amplicons were subjected to paired-end read sequencing using Illumina MiSeq (v2, 300-cycle). Rare sequence reads that occur only once were excluded to remove errors associated with sequencing reaction and amplification. Insertions or deletions located around the CRISPR/Cas9 cleavage site (3 bp upstream of the PAM) were considered to be the mutations induced by CRISPR/Cas9.

### Quantitative real-time PCR

Total RNA was extracted using the easy-spin Total RNA Extraction Kit (iNtRON, Seoul, Korea), according to the manufacturer’s protocol, and the total RNA concentration was quantified using a NanoDrop 2000 Spectrophotometer (Thermo Fisher Scientific, Wilmington, DE, USA). The cDNA was synthesized using Maxime RT Premix (iNtRON) according to the manufacturer’s protocol. A PCR plate (MicroAmp optical 96-well reaction plate) was made by adding 1 μL cDNA, 0.4 μL (10 pmol/μL) forward primer, 0.4 μL (10 pmol/μL) reverse primer, 10 μL SYBR Premix Ex Taq (Takara, Otsu, Japan) and 8.2 μL of Nuclease-free water (Ambion, Austin, TX, USA). The reactions were carried out for 40 cycles and the cycling parameters were as follows: denaturation at 95 °C for 15 s, annealing at 60 °C for 1 min and extension at 72 °C for 1 min. All oligonucleotide primer sequences are presented in Table 2. The expression of each target gene was quantified relative to that of the internal control gene (*GAPDH*) using the equation, R = 2^-[ΔCt sample - ΔCt control]^.

**Table 2:** List of real-time PCR primers

Gene	Primer sequences (5′-3′)		Product size (bp)	GenBank accession number
	Forward	Reverse		
*GAPDH*	GTCGGTTGTGGATCTGACCT	TTGACGAAGTGGTCGTTGAG	207	NM_001206359
*IGF1*	AGGAGGCTGGAGATGTACTG	TGGCATGTCATTCTTCACTC	191	NM_214256
*IGF1R*	ATTCGCACCAATGCTTCA	AGGGCGGGTTCCACTTC	94	NM_214172
*FOXO1*	CATTGAGCGCTTAGACTGTG	TCTCAGTTCCTGCTGTCAGA	214	NM_214014
*MnSOD*	GCTTACAGATTGCTGCTTGT	AAGGTAATAAGCATGCTCCC	101	NM_214127
*CAT*	TTAATCCATTCGATCTCACC	GGCGGTGAGTGTCAGGATAG	210	NM_214301
*BAX*	TGCCTCAGGATGCATCTACC	AAGTAGAAAAGCGCGACCAC	199	XM_003127290
*BCL2*	AGGGCATTCAGTGACCTGAC	CGATCCGACTCACCAATACC	193	NM_214285
*CASPASE3*	CGTGCTTCTAAGCCATGGTG	GTCCCACTGTCCGTCTCAAT	186	NM_214131

### Western blot analysis

Fetal fibroblasts and placentas were finely grinded by homogenizer and washed with PBS and lysed with ice-cold lysis buffer (50 mM Tris-HCl, pH 7.2; 150 mM NaCl; 1 mM EDTA; 1% Triton X-100; 0.1% aprotinin; 0.1% SDS; 1 mM PMSF). The lysate was clarified by centrifugation (13,000 rpm, 10 min at 4 °C). Equal amounts of proteins from the supernatant was fractionated by 10% SDS-PAGE and electrotransferred onto a PVDF membrane. The blots were blocked in 5% skim milk at 4 °C overnight. In the next day, the membrane was incubated with rabbit anti-klotho antibody (1:1000, Abcam, Cambridge, UK) and rabbit anti-β-actin (1:5000, Abcam) diluted with 5% skim milk Tris-buffered saline containing 0.1% Tween 20 (TBST) for 2 h. Then membranes were washed three times each for 10 min with TBST incubated with horseradish peroxidase-conjugated goat anti-rabbit IgG (1:5000) for 1 h. The blots were developed using the Pierce SuperSignal West Pico Chemiluminescent System (Thermo Fisher Scientific). The densities of the immunoblots were scanned with image acquisition system (Fusion SL3500; Vilber Lourmat, Eberhardzell, Germany).

### Off-target analysis

Ten potential off-target sites (OTSs) for the sgRNA targeting porcine *klotho* gene were predicted using the Cas-OFFinder online tool (http://www.rgenome.net/cas-offinder/) to analyze site-specific cleavage by the CRISPR/Cas9 system. All the potential OTSs were PCR amplified and their PCR products were sequenced using an ABI 3730XL DNA analyzer (Applied Biosystems, CA, USA) to confirm the off-target events. Sequencing data were analyzed using a Variant Reporter Software Version 2.0 (Applied Biosystems). Sequences of the potential OTSs are listed in Additional file 1: Table S1 and primers used for OTSs determination were listed in Additional file 1: Table S2.

### Statistical analysis

Statistical analyses were performed using SPSS 22.0 (SPSS, Inc., IL, USA). All data were tested for normality and homoscedasticity, then subjected to a Mann-Whitney’s *U*-test for data with non-normal distribution or Student’s *t*-test for data with normal distribution to determine differences between experimental groups. Data are expressed as means ± SEM. *P* values < 0.05 were considered to be statistically significant.

## Supplementary Information

**Additional file 1: Table S1.** Summary of potential off-target sites (OTSs) for the sgRNA targeting porcine *klotho* gene. **Table S2.** Primer sequences of potential off-target sites (OTSs) for the sgRNA targeting porcine *klotho* gene.

**Additional file 2: Figure S1.** Uncropped gel images for Fig. 1d. T7 endonuclease I (T7E1) assay: the T7E1 assay was conducted using genomic DNA from 20 blastocysts cloned from non-selected porcine fibroblasts transfected with Cas9-sgRNA RNPs and 9 single colonies of porcine fibroblasts transfected with Cas9-sgRNA RNPs. (M, Marker; WT, wild-type; PC, positive control; #, single colony cell line; BL, blastocyst). **Figure S2.** Uncropped immunoblot images for Fig. 3e. Expression of klotho protein between *klotho* monoallelic knockout and wild-type placentas detected by Western blot analysis. WT, wild-type; Fetus V2; viable fetus 2 (WT/− 17 bp,+ 12 bp). **Figure S3.** Uncropped immunoblot images for Fig. 4e. Expression of klotho protein between *klotho* monoallelic knockout and wild-type placentas detected by Western blot analysis. WT, wild-type; Fetus V2; viable fetus 2 (WT/− 17 bp,+ 12 bp).

## Data Availability

The datasets used and/or analyzed during the current study are available from the corresponding author on reasonable request.

## Abbreviations

IGF1: Insulin-like growth factor 1
IGF1R: Insulin-like growth factor 1 receptor
FOXO1: Forkhead box O1
MnSOD: Manganese-dependent superoxide dismutase
CAT: Catalase
BCL2: B cell leukemia/lymphoma 2
BAX: BCL2 associated X
