# Correction to: Failure to maintain full-term pregnancies in pig carrying klotho monoallelic knockout fetuses

**DOI:** 10.1186/s12896-021-00671-0

**Published:** 2021-02-02

**Authors:** Sanghoon Lee, Min Hee Jung, Kilyoung Song, Jun-Xue Jin, Anukul Taweechaipaisankul, Geon A. Kim, Hyun Ju Oh, Ok Jae Koo, Se Chang Park, Byeong Chun Lee

**Affiliations:** 1 Department of Theriogenology and Biotechnology, College of Veterinary Medicine, Seoul National University, Seoul, Republic of Korea; 2 Futuristic Animal Resource & Research Center, Korea Research Institute of Bioscience and Biotechnology, Cheongju-si, Chungcheongbuk-do Republic of Korea; 3 Toolgen, Inc., Seoul, Republic of Korea; 4 Key Laboratory of Animal Cellular and Genetic Engineering of Heilongjiang Province, College of Life Science, Northeast Agricultural University, Harbin, Heilongjiang China; 5 Department of Biomedical Laboratory Science, School of Medicine, Eulji University, Daejeon, Republic of Korea; 6 Laboratory of Aquatic Biomedicine, College of Veterinary Medicine and Research Institute for Veterinary Science, Seoul National University, Seoul, Republic of Korea

**Correction to: BMC Biotechnol (2021) 21:1**

**https://doi.org/10.1186/s12896-020-00660-9**

Following publication of the original article [1], the authors informed us that the author Se Chang Park was incorrectly affiliated.

The incorrect affiliation is:

5Department of Biomedical Laboratory Science, School of Medicine, Eulji University, Daejeon, Republic of Korea.

6Laboratory of Aquatic Biomedicine, College of Veterinary Medicine and Research Institute for Veterinary Science, Seoul National University, Seoul, Republic of Korea.

The correct affiliation is:

6Laboratory of Aquatic Biomedicine, College of Veterinary Medicine and Research Institute for Veterinary Science, Seoul National University, Seoul, Republic of Korea.

Also, the authors identified an error in the Additional file 2: Figure S2.

The sentence currently reads:

"Figure S2. Uncropped immunoblot images for Fig. 3e. Expression of klotho protein between klotho monoallelic knockout and wild-type **placentas** detected by Western blot analysis. WT, wild-type; Fetus V2; viable fetus 2 (WT/− 17 bp,+ 12 bp). Figure S3. Uncropped immunoblot images for Fig. 4e. Expression of klotho protein between klotho monoallelic knockout and wild-type placentas detected by Western blot analysis. WT, wild-type; Fetus V2; viable fetus 2 (WT/− 17 bp,+ 12 bp)."

The sentence should read:

"Figure S2. Uncropped immunoblot images for Fig. 3e. Expression of klotho protein between klotho monoallelic knockout and wild-type **fibroblasts** detected by Western blot analysis. WT, wild-type; Fetus V2; viable fetus 2 (WT/− 17 bp,+ 12 bp). Figure S3. Uncropped immunoblot images for Fig. 4e. Expression of klotho protein between klotho monoallelic knockout and wild-type placentas detected by Western blot analysis. WT, wild-type; Fetus V2; viable fetus 2 (WT/− 17 bp,+ 12 bp)."

The errors were introduced during the production process. The publisher apologizes for any confusion.

The original article [1] has been corrected.
