# Supplementary material for: Failure to maintain full-term pregnancies in pig carrying klotho monoallelic knockout fetuses
Source: BMC Biotechnol. 2021 Jan 7;21:1. doi: 10.1186/s12896-020-00660-9 (PMC7791653; doi:10.1186/s12896-020-00660-9)
Supplement: Supplementary file 1 — Additional file 1: Table S1. Summary of potential off-target sites (OTSs) for the sgRNA targeting porcine klotho gene. Table S2. Primer sequences of potential off-target sites (OTSs) for the sgRNA targeting porcine klotho gene. [file 12896_2020_660_MOESM1_ESM.docx]

**Table S1** Summary of potential off-target sites (OTSs) for the sgRNA targeting porcine *klotho* gene

| Name | Sequence^a,b^ | Location on chromosome | Position | Direction | Mismatches | Bulge size | Effect |
| --- | --- | --- | --- | --- | --- | --- | --- |
| OTS1 | TGAGAACAAGGCaGAAGcCTTTGG | AEMK02000677.1 | 1190946 | + | 2 | 1 | None |
| OTS2 | TGAGAACAAGGCaGAAGcCTTGGG | CM000820.5 | 13057103 | - | 2 | 1 | None |
| OTS3 | TAGcAgAAGGGCTGAAGACTTGGG | CM000825.5 | 16034675 | + | 2 | 1 | None |
| OTS4 | aAGAACAAGGCTGATAGAtTTGGG | CM000815.5 | 68625512 | - | 2 | 1 | None |
| OTS5 | cAGAGAaAAGGCTGAAGACTTAGG | CM000826.5 | 104424507 | + | 2 | 1 | None |
| OTS6 | TAGACAgAAGGgTGAAGACTTTGG | CM000820.5 | 106664481 | + | 2 | 1 | None |
| OTS7 | TCAGtACAAGGCTGAtGACTTGGG | CM000824.5 | 174790482 | - | 2 | 1 | None |
| OTS8 | TAGACCACAAGGCTGAAGAtTTGGG | CM000812.5 | 245124708 | - | 1 | 2 | None |
| OTS9 | cAGAACAAGGCAAGACTTAGG | CM000813.5 | 104692006 | - | 1 | 2 | None |
| OTS10 | TAGAACAAaGGAAGACTTTGG | CM000830.5 | 90794433 | + | 1 | 2 | None |

^a^ Lower letters indicate the different nucleotides from on-target site.

^b^ NGG indicates PAM sequence.

**Table S2** Primer sequences of potential off-target sites (OTSs) for the sgRNA targeting porcine *klotho* gene

| Name | Primer sequences (5’-3’) | | Product size (bp) |
| --- | --- | --- | --- |
|  | Forward | Reverse |  |
| OTS1 | GTCTCCATCACTTGCCTTTCCAAGTAGAAGACC | GAAGAAGCAGAACACAAAGATGTCAGAACTGG | 605 |
| OTS2 | CAGGTGCGCAGGAGCAGAGCACAAAGTGAG | CCTGCTGACCTCGGTGGGACAGATCTGACC | 595 |
| OTS3 | GCCTTCACAGATGTGAATGCTATGTATGATAAGG | CATCTGATCAGCATTATTGCTACCTTCACACC | 590 |
| OTS4 | CCAGCTGTGACGTCCCTGCACAAGTCCTGTTC | GTTGGAAGTGAGGTAAGAGTTCGGGAGGAGG | 582 |
| OTS5 | GTTAATGAACCCGACTAGGAACCATGAAGTTAAGG | GGCACATCTGTCTTCCTGGGTTATAACAGATCC | 588 |
| OTS6 | CTAACGTCTTGGCTGATGTCTACATGTCACC | GGAGCTTCCATGAGAGTCAAGGCAGCCAAAC | 333 |
| OTS7 | CCATCAAGTAGGAAAGAAGCAGACTGCCTAGGTG | GTATCCAGCCTGTGTAGAGAAGAGACTTCAGAC | 797 |
| OTS8 | GGTTAGTGTGAACTGACCCAAATAATTCCAC | CCAGCATGTACACTAGTCTCTTATACATGAAGG | 598 |
| OTS9 | CGAATGGACTTTAGCCTAAAATAATCCATATGCC | CAGCATCTTCTGTTGTTCTAGTAATTTCATTGC | 600 |
| OTS10 | CATGGAAGGAAGGGATAAGAAGCGATGGATCC | GGCTAGGGTAGTTATTACATAGAGCATCTGGAG | 603 |
