# Supplementary material for: Failure to maintain full-term pregnancies in pig carrying klotho monoallelic knockout fetuses
Source: BMC Biotechnol. 2021 Jan 7;21:1. doi: 10.1186/s12896-020-00660-9 (PMC7791653; doi:10.1186/s12896-020-00660-9)
Supplement: Supplementary file 2 — Additional file 2: Figure S1. Uncropped gel images for Fig. 1d. T7 endonuclease I (T7E1) assay: the T7E1 assay was conducted using genomic DNA from 20 blastocysts cloned from non-selected porcine fibroblasts transfected with Cas9-sgRNA RNPs and 9 single colonies of porcine fibroblasts transfected with Cas9-sgRNA RNPs. (M, Marker; WT, wild-type; PC, positive control; #, single colony cell line; BL, blastocyst). Figure S2. Uncropped immunoblot images for Fig. 3e. Expression of klotho protein between klotho monoallelic knockout and wild-type placentas detected by Western blot analysis. WT, wild-type; Fetus V2; viable fetus 2 (WT/− 17 bp,+ 12 bp). Figure S3. Uncropped immunoblot images for Fig. 4e. Expression of klotho protein between klotho monoallelic knockout and wild-type placentas detected by Western blot analysis. WT, wild-type; Fetus V2; viable fetus 2 (WT/− 17 bp,+ 12 bp). [file 12896_2020_660_MOESM2_ESM.pptx]

## Slide 1
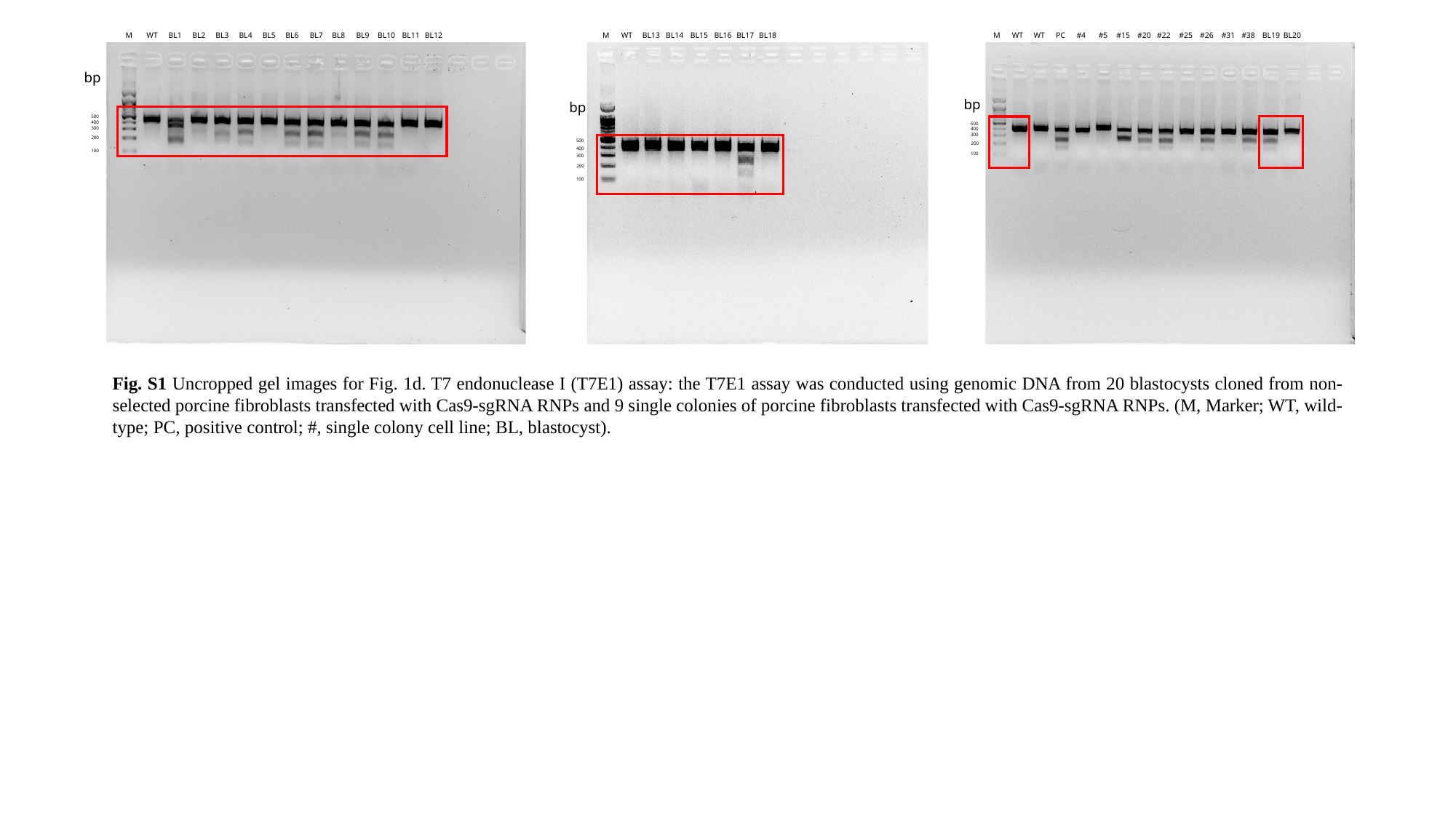

M
WT
BL1
BL2
BL3
BL4
BL5
BL6
BL7
BL8
BL9
BL10
BL11
BL12
M
WT
BL13
BL14
BL15
BL16
BL17
BL18
M
WT
WT
PC
#4
#5
#15
#20
#22
#25
#26
#31
#38
BL19
BL20
bp
bp
bp
500
400
500
300
400
300
200
500
200
400
100
100
300
200
100
Fig. S1 Uncropped gel images for Fig. 1d. T7 endonuclease I (T7E1) assay: the T7E1 assay was conducted using genomic DNA from 20 blastocysts cloned from non-selected porcine fibroblasts transfected with Cas9-sgRNA RNPs and 9 single colonies of porcine fibroblasts transfected with Cas9-sgRNA RNPs. (M, Marker; WT, wild-type; PC, positive control; #, single colony cell line; BL, blastocyst).

## Slide 2
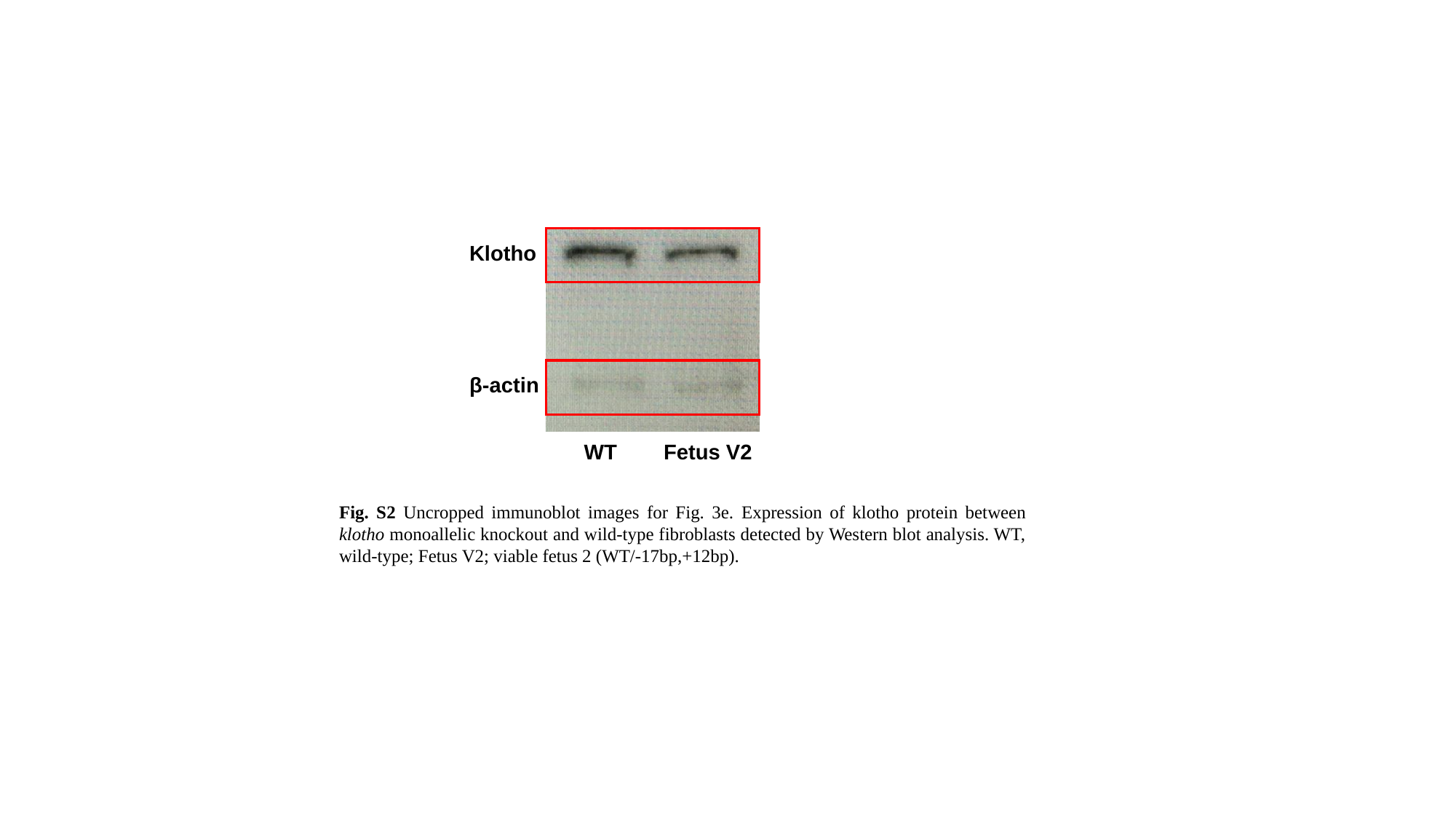

Klotho
β-actin
Fetus V2
WT
Fig. S2 Uncropped immunoblot images for Fig. 3e. Expression of klotho protein between klotho monoallelic knockout and wild-type fibroblasts detected by Western blot analysis. WT, wild-type; Fetus V2; viable fetus 2 (WT/-17bp,+12bp).

## Slide 3
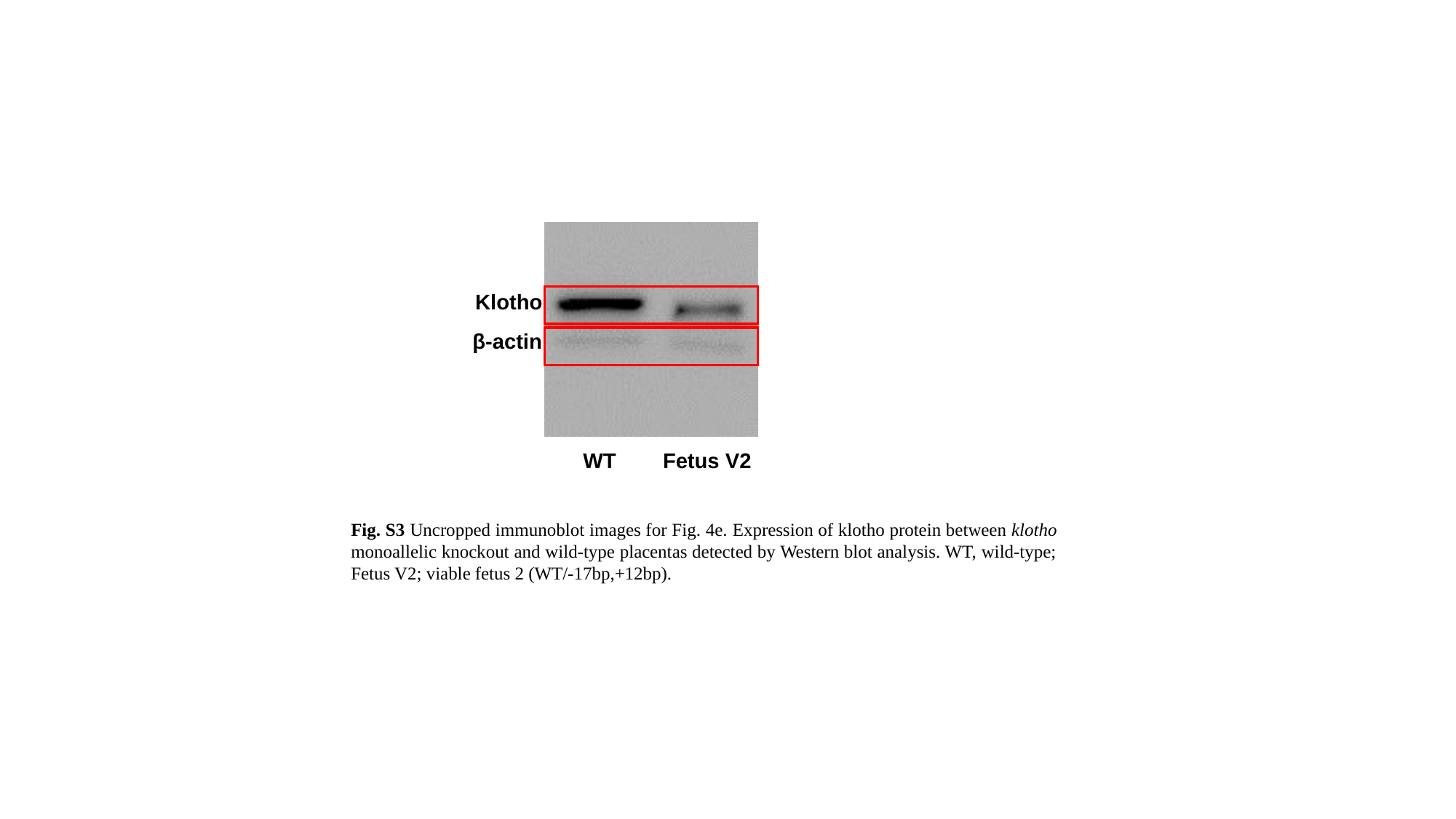

Klotho
β-actin
WT
Fetus V2
Fig. S3 Uncropped immunoblot images for Fig. 4e. Expression of klotho protein between klotho monoallelic knockout and wild-type placentas detected by Western blot analysis. WT, wild-type; Fetus V2; viable fetus 2 (WT/-17bp,+12bp).
